# Master regulator: p53’s pivotal role in steering NK-cell tumor patrol

**DOI:** 10.3389/fimmu.2024.1428653

**Published:** 2024-08-09

**Authors:** Haohao Wang, Qingjie Chen, Qinghua Liu, Changjiang Luo

**Affiliations:** Department of General Surgery, Lanzhou University Second Hospital, Lanzhou, China

**Keywords:** p53, NK cells, tumor microenvironment, tumor surveillance, immunotherapy

## Abstract

The p53 protein, encoded by TP53, is a tumor suppressor that plays a critical role in regulating apoptosis, cell cycle regulation, and angiogenesis in tumor cells via controlling various downstream signals. Natural killer (NK) cell-mediated immune surveillance is a vital self-defense mechanism against cancer and other diseases, with NK cell activity regulated by various mechanisms. Among these, p53 plays a significant role in immune regulation by maintaining the homeostasis and functionality of NK cells. It enhances the transcriptional activity of NK cell-activating ligands and downregulates inhibitory ligands to boost NK cell activation and tumor-killing efficacy. Additionally, p53 influences NK cell cytotoxicity by promoting apoptosis, autophagy, and ferroptosis in different tumor cells. p53 is involved in the regulation of NK cell activity and effector functions through multiple pathways. p53 also plays a pivotal role in the tumor microenvironment (TME), regulating the activity of NK cells. NK cells are critical components of the TME and are capable of directly killing tumor cells. And p53 mutates in numerous cancers, with the most common alteration being a missense mutation. These mutations are commonly associated with poor survival rates in patients with cancer. This review details p53’s role in NK cell tumor immunosurveillance, summarizing how p53 enhances NK cell recognition and tumor destruction. We also explore the potential applications of p53 in tumor immunotherapy, discussing strategies for modulating p53 to enhance NK cell function and improve the efficacy of tumor immunotherapy, along with the associated challenges. Understanding the interaction between p53 and NK cells within the TME is crucial for advancing NK cell-based immunotherapy and developing p53-related novel therapeutics.

## 1 Introduction

Natural killer (NK) cells play an essential role in the innate immune system as they are involved in the first line of defense against viral infection and aberrant cells. Not only do they possess the unique capability to lyse virus-infected and malignant cells directly, but they also carry out diverse functions (1). In humans, NK cells are divided into two main subpopulations based on their Fcγ receptor III CD56 and CD16 expression: the CD56^dim^CD16^high^ and CD56^bright^CD16^low/−^ populations (2). The CD56^bright^CD16^low/−^ NK cells are the major producers of cytokines, particularly interferon-gamma (IFN-γ). The CD56^dim^CD16^high^ NK cells exhibit potent cytotoxic activity and are relatively deficient in IFN-γ secretion (3). When NK cells identify target cells, they release cytotoxic particles, leading to direct lysis of the target cells while also producing immunomodulatory factors such as IFN-γ, tumor necrosis factor-alpha (TNF-α) and interleukin 12 (IL-12) (4, 5). Furthermore, NK cells exert their cytotoxic effects by expressing death receptor ligands and transporting the granzyme serine protease (6, 7). In tumor cells, NK cells can either directly kill the tumor cells through their cytotoxicity or indirectly destroy them through the secretion of cytotoxic mediators such as granzyme B(GzmB) and perforin (8). Numerous clinical trials are currently underway to evaluate the effectiveness of NK cell-based immunotherapies, including chimeric antigen receptor-modified NK cells (CAR-NK), the immune checkpoint inhibitor lirilumab, and others. However, the results of these trials are still pending evaluation (9, 10). In this situation, it is of great importance to gain an understanding of the intricate processes that enhance the tumor-killing potential of NK cells. The p53 protein, which is encoded by the human gene TP53, plays a pivotal role in many cancers, regulating the interaction between tumors and NK cells.

p53 is a crucial and influential transcription factor, the DNA-binding domain of p53 binds to the promoter elements of the corresponding regulatory genes. It initiates the expression of the corresponding genes, thereby initiating regulatory pathways, such as p21, Bax, and FASL (11). The coordination of target gene expression by p53 contributes significantly to cell cycle regulation, DNA repair, and apoptosis (12). In NK cells, the tumor suppressor protein p53 affects NK cell function by regulating the cell cycle and apoptosis, which are influenced by these pathways (13). The wild-type p53 (wtp53) has been described as a tumor suppressor that inhibits tumor cell proliferation and angiogenesis. Furthermore, activation of the p53 pathway has been observed to promote apoptosis in cancer cells, enhance antigen presentation, and may stimulate tumor killing by NK cells by promoting the expression of activating ligands (14, 15). Furthermore, p53 exerts its influence through “non-canonical” mechanisms, such as regulating cellular autophagy, metabolism, senescence, and iron death (16, 17). During the process of tumorigenesis, tumor cells have the capacity to significantly influence surrounding cells, thereby creating a tumor microenvironment (TME) that serves to facilitate cancer progression. In cancer cells that express wtp53, regulation of the p53 pathway may enhance tumor immunogenicity for NK cells, generate an inflammatory TME, and ultimately lead to tumor regression (18). In TME, p53 is also involved in the regulation of immune cells, including T cells and macrophages. For instance, p53 can enhance anti-tumor immune responses by regulating the polarization state of macrophages and promoting the activity of M1-type macrophages. Furthermore, p53 can regulate the function of T cells and enhance the overall anti-tumor immune response by promoting the activation and proliferation of T cells (19, 20). Moreover, these immune cells can interact with NK cells, thereby jointly suppressing tumor progression (21, 22). It is noteworthy that in tumor cells, the TP53 gene is somatically mutated in more than half of cancer cases. More than 75% of these TP53 gene alterations are missense mutations (mutp53), which encode full-length but dysfunctional mutant proteins (23). P53-based therapy has been demonstrated to enhance the function of NK cells in tumor immune surveillance. Early studies have indicated that the immune mechanism of NK cells plays a crucial role during adenoviral p53 gene therapy (24). Furthermore, p53 activation has been demonstrated to enhance the sensitivity of immune checkpoints, as well as other novel NK cell-based therapeutic strategies, to treat tumors (25, 26). This review provides a comprehensive overview of the pivotal role played by p53 in regulating NK cell activity and tumor immune surveillance, while also summarizing the latest advancements in therapeutic strategies and addressing challenges associated with utilizing p53 to augment NK cell function for enhanced tumor eradication. This contribution enhances our understanding of the molecular mechanisms underlying NK cell-mediated immune surveillance and facilitates the exploration of novel approaches to combat cancer.

## 2 The multifaceted role of p53 in tumor immune surveillance by NK cells

### 2.1 Regulatory role of p53 in NK cell

The balance of cell cycle and apoptosis is crucial for the proliferation and activation of NK cells. p53 is a potential regulator of NK cell functional maturation. p53-related pathways, such as the p21/p53 apoptosis pathway and the p53/CDK cyclic regulatory pathway, play an important role in maintaining the maturation process of NK cells during the process of NK cell maturation (27, 28). Due to the tight regulation of NK cells by the cell cycle, Karlhofer FM (29) et al. were unable to consistently extract proliferating NK cells from mice with the wtp53 gene. However, they were able to extract them from mice with a deletion of the p53 gene, which indicates that p53 plays a critical role in maintaining a normal cell cycle and preventing aberrant proliferation of NK cells. p53 plays a pivotal role in the regulation of cellular metabolism as a chaperone protein for HIF-1α. In the hypoxic environment, HIF-1α is capable of interacting directly with p53 (30). In this context, the metabolic shifts induced by HIF-1α in NK cells are essential for overcoming hypoxia-mediated impairments in critical functions such as survival, proliferation, and tumor cytotoxicity. Additionally, p53 can respond to these metabolic changes. In NK cells, p53-targeted cell cycle arrest proteins, such as CDKN1A, GADD45A, and MDM2, are down-regulated, while cell cycle proteins like CCNE1 and CCNF, as well as cell division proteins such as CDC6 and CDC20, are up-regulated. Furthermore, p53-regulated apoptosis-related proteins are also down-regulated (13). When NK cells are stimulated by radiotherapy and proliferation disorders, they activate the DNA damage response (DDR) through ATM phosphorylation. For example, when CD56^bright^CD16^low/-^NK cells are stimulated by UV light and DNA damage occurs, ATM phosphorylation and p53 expression in NK cells are increased, leading to cell cycle arrest and increased apoptosis (31). While investigating the role of p53 in natural killer T-cell lymphoma (NKTCL) tumors, some researchers found that p53 was negatively correlated with PLK1 expression in NKTCL tissues as well as in the NK92 cell line and that downregulation of PLK1 initiated the ATM/p53 signaling pathway, which induced DDR and thus facilitated apoptosis in cancerous NK cells (32). p53 is also involved in regulating the expression of multiple receptors on the surface of NK cells, which serve as crucial mediators of NK cell activation and function. It has been demonstrated that inhibition of p53 followed by reactivation significantly enhances the expression of B220 markers on NK cells in the context of lung adenocarcinoma TME. NK cells expressing B220 receptors exhibit heightened tumor-killing activity, accompanied by the production of large quantities of IFN-γ and other cytokines. Furthermore, IFN-γ overexpression has been demonstrated to enhance the capacity of MHC-I to present antigens (33). In addition to directly regulating activating or inhibitory receptors on the surface of NK cells to enhance their activity, p53 also interacts with signaling molecules downstream of these receptors to promote NK cell activation and proliferation. Signal-regulatory protein (SAP), a junction molecule with an SH2 structural domain, is expressed in activated NK cells and T cells. SAP binds to the signaling lymphocytic activation molecule (SLAM) receptor on the cell surface, promoting NK cell activation and exerting its cytotoxic effects. In NK cells, p53 is a transcription factor for SAP that directly interacts with SAP and upregulates SAP expression, while SAP couples the SLAM receptor to the kinase Fyn, which activates the exchange factor Vav-1 and enhances NK cell activation (34, 35). These roles of p53 in NK cells lay the foundation for NK cell recognition and targeting of tumor cells.

### 2.2 Regulatory role of p53 in tumor cells

#### 2.2.1 Regulation of NK cell receptor ligands

In the immune system, the activation of NK cells is a complex and delicate process, and their effector function is mainly controlled by the action of surface-activating and inhibitory ligands with them. The balance of signals transmitted through these receptors is of utmost importance in the maintenance of immune system homeostasis (36). NK cell inhibitory ligands mainly include histocompatibility complex class I (MHC I) molecules (37), and activating ligands such as MICA, ULBP1, ULBP2, and poliovirus receptor (PVR) mainly interact with NKG2D and DNAM-1 (26, 38). P53 plays a primary role in the tumor surveillance and elimination functions of NK cells. This is achieved by up-regulating the expression of NK cell-activating ligands and down-regulating inhibitory ligands. Consequently, NKG2D is essential in NK cell-mediated tumor surveillance and is expressed mainly on NK cells and T cells. The binding of NKG2D to these ligands directly induces the activation of NK cells (39–41). Studies have demonstrated that the wtp53 protein in the non-small cell lung cancer (NSCLC) cell line H1299P53 is capable of regulating the ligands ULBP1 and ULBP2, ligands for the NKG2D receptor on NK cells. This regulation involves the up-regulation of their expression. Furthermore, their studies have demonstrated that p53 deletion not only promotes NSCLC cell proliferation but also leads to a decrease in the expression of NKG2D ligands, rendering cancer cells more likely to evade recognition by NK cells and undergo immune evasion (15). Similar effects have been described in multiple myeloma (MM) cell lines, the expression of ULBP1 in MM cells can be attributed to the increased transcriptional activity of ULBP1, which is mediated by the activation of ATM/p53 of the DDR-related pathway and its enhanced mRNA stability (42). Hai Li (43) et al. discovered that p53 does not rely on the DDR signaling pathway ATM/ATR to promote ULBP2 expression in colorectal cancer HCT116 and breast cancer MCF7 cell lines. Their research demonstrated that p53 induces ULBP2 expression by inhibiting DNA methyltransferase (DNMT), facilitating the demethylation of the p53-binding region within the ULBP2 gene. Furthermore, in melanoma cells, the WEE1/AKT pathway was found to mediate the transcriptional regulation of ULBP1 and ULBP2 by p53 (18). Additionally, p53 regulates the expression of the NKG2D-activating ligand, Mult-1, on cancer cells, which in turn activates the production of IFN-γ by NK cells in the breast cancer EMT6 cell line (44). DNAM-1 is another receptor for NK cells, and PVR is an activating ligand for DNAM-1. p53 facilitates the pathway for NK cells to recognize and attack tumor cells by promoting the interaction of ligands such as PVR and Neuroendocrine Convertase-2 (Nec2) with DNAM-1 (45). In neuroblastoma cells, p53 can bind to the PVR promoter and directly regulate PVR expression, thereby promoting PVR/DNAM-1 interaction and enhancing the recognition and killing of neuroblastoma by NK cells (46). Moreover, Nec2 has been identified as another activating ligand for DNAM-1 on NK cells, which plays a crucial role in recognizing and eliminating adult acute lymphoblastic leukemia stem cells (47). Regulation of Nec2 expression by p53 was also observed in melanoma cells, which was also dependent on the activation of p53 by the WEE1/AKT signaling axis (18). In MM cells, p53 also promotes the expression of intercellular adhesion molecule-1 (ICAM-1) on its surface, which facilitates the binding of ICAM-1 to its receptor, lymphocyte function-associated antigen 1 (LFA-1). This binding enables ICAM-1/LFA-1 to activate NK cell-mediated cytotoxicity and enhance the antitumor effects of NK cells. Furthermore, ICAM-1 expression in tumor cells is essential for perforin-mediated cancer cell lysis by NK cells (48–50). In addition, p53 also regulates the expression of inhibitory ligands. Pharmacological activation of p53 in the colorectal cancer HCT116 cell lines and the breast cancer MCF7 cell lines has been observed to inhibit the expression of the MHC I molecules HLA-A, B, and C. It has been shown that the reduced expression of MHC I molecules promotes the killing of target cells by NK cells (43). In addition to upregulating the activating ligand Mult-1, p53 also downregulates the expression of the inhibitory ligand H60a in the breast cancer EMT6 cell lines. This is an interesting finding, as H60a is an inhibitory ligand for NKG2D (44). B7-H3 acts as an immune checkpoint molecule and its overexpression can impede the recognition and elimination of tumors by NK cells (51). It has been demonstrated that loss of p53 leads to activation of the transcription factor Sp1, resulting in upregulated expression of B7-H3 and suppression of immune cell infiltration, particularly NK cells. This process has been associated with prostate cancer progression (25). Collectively, p53 exerts a fine regulatory influence on the expression of NK cell ligands through a multiplicity of mechanisms, including transcriptional activation, modulation of the DNA methylation status, and interaction with signaling axes. Together, these mechanisms constitute a complex regulatory network that affects the activation state of NK cells and the efficiency of tumor immunosurveillance. Furthermore, the regulation of these ligand expressions by p53 enriches our understanding of the role of NK cells in tumor immunosurveillance.

#### 2.2.2 Role of p53 in NK cell-induced tumor apoptosis

The induction of mitochondrial apoptosis is a complex process involving multiple steps. NK cells can induce mitochondrial apoptosis in cancer cells (52). NK cell-mediated apoptosis in target cells primarily occurs through the release of cleavage particles and the binding of death receptors to ligands (53). The effectiveness of this process depends on the state of mitochondrial initiation. It has been demonstrated that BH3, an inhibitor of the anti-apoptotic protein Bcl-2, is capable of limiting the mitochondrial initiation state. Furthermore, BH3 can be utilized in conjunction with NK cells to enhance the efficacy of NK-cell-based immunotherapy for the treatment of cervical cancer (54). p53 plays a pivotal role in NK cell-mediated apoptosis in the breast cancer cell line MCF7. p53 is capable of binding to Bcl-2 and participating in the granzyme B (GzmB)-mediated apoptotic pathway, thereby preventing the interaction of truncated Bid (tBid), produced by GzmB. The inhibition of this interaction results in the activation of Bax by truncated Bid at the mitochondrial membrane, which facilitates the GzmB-induced increase in mitochondrial outer membrane permeability, thereby expediting the pro-apoptotic effect of NK cells on tumor cells (55). In addition, the small molecule CP-31398 was found to be able to reactivate wtp53 in mutp53 breast cancer and enhance GzmB -mediated apoptosis in NK cells via the sestrin/AMPK/mTOR axis and ULK-1 (16). Granzyme K (GzmK) is expressed at high levels in NK cells, the initial study demonstrated that GzmK could cleave p53 into p40, p35, and p13 fragments by interacting with p53. These three cleavage products were found to amplify the pro-apoptotic effects of p53 in NK cells and enhance the pro-apoptotic effects of NK cells on tumors (56). GzmK may also interact with p53 to promote apoptosis of tumor cells indirectly through Apurinic apyrimidinic endonuclease (APE1), which is a substrate of GzmK. GzmK is also able to promote the production of reactive oxygen species to promote apoptosis of target cells through the degradation of APE1 (57). While APE1 can interact with p53 to promote p53 degradation, inhibition of APE1 can upregulate p53 to promote apoptosis in lung adenocarcinoma cells (58). In responding to apoptosis mediated by granzyme secreted by NK cells, p53 also participates in apoptosis mediated by interactions between NK cells and tumor cell death receptor ligands. This indirectly enhances tumor-killing by NK cells by regulating the expression of the death receptor on the surface of tumor cells. FasL, a death receptor and target of p53 expressed on the surface of many different cell types, can induce apoptosis upon binding to ligands (59). This ligand-induced apoptosis can be observed in a variety of cell types. NK cells exert their cytotoxic effects primarily through the Fas/FASL pathway (60). A study on the effects of polysaccharide (DLP120) indicated that DLP120 was able to promote NK cell activity in a mouse model of hepatocellular carcinoma and promoted the p53-mediated Fas/Fasl pathway, thereby promoting apoptosis in hepatocellular carcinoma cells (61). Tumor necrosis factor-associated apoptosis-inducing ligand (TRAIL) is another p53 target gene, which is mainly expressed on the surface of immune cells such as NK cells and T cells (62). The interaction between TRAIL on the surface of NK cells and TRAILR1/TRAILR2 on the surface of colon cancer cells initiates an exogenous apoptosis pathway. Furthermore, the expression of TRAILR1/TRAILR2 on the surface of colorectal cancer cells is up-regulated by p53 to promote apoptosis. TRAILR2 expression has been demonstrated to facilitate NK cell-mediated apoptosis in colorectal cancer cells and augment the sensitivity of NK cell activity (63). It is notable that p53 only upregulated the expression of TRAILR2 and thus sensitivity to TRAIL receptors in myeloma cells, while not increasing the expression of TRAILR1 (53).

#### 2.2.3 Additional mechanisms of p53 regulation in NK cell-mediated tumor surveillance

p53 also affects the tumor-killing effect of NK cells by participating in the iron death and autophagy pathways. Research has demonstrated that p53 is capable of inducing iron-mediated cell death in tumor cells, including those of ovarian cancer. Furthermore, the iron-mediated cell death of tumor cells has been shown to result in the recruitment of a significant number of NK cells, which can then facilitate the clearance of tumors by NK cells (64, 65). Studies have identified that p53 regulates ferroptosis processes within extra-nodal NK/T-cell lymphoma (ENKTCL) cells by affecting GSH synthesis by inhibiting SLC7A11 expression, leading to reduced GPX4 activity and subsequent ferroptosis induction (66) The pathway involving p53/SLC7A11/GPX4 also plays a role in ferroptosis in cholangiocarcinoma cells. However, this investigation did not establish a connection between p53-mediated ferroptosis in cholangiocarcinoma cells and the function of NK cells. Additionally, research has indicated that p53 is implicated in a non-GPX4-dependent ferroptosis pathway. Specifically, dihydroartemisinin induced ferroptosis in the pancreatic cancer cell lines Panc02 and Panc1 through a p53/ALOX12-dependent mechanism and further facilitated the recruitment of NK cells into the tumor tissues of a mouse model of *in situ* pancreatic cancer (67). Chollet et al. (16) demonstrated that in breast cancer, p53 can promote the autophagy of tumor cells through AMPK activation. Furthermore, mTOR inhibition enhances the susceptibility of NK cells to tumor lysis. Studies have demonstrated that p53 is involved in the autophagy process in ENKTCL. Inhibition of the p53/mTOR signaling pathway has been shown to promote autophagy, leading to drug resistance in ENKTCL. Conversely, p53 is involved in inhibiting the autophagy process within ENKTCL (68). This is in contrast to the role of p53 in breast cancer autophagy, which is due to the dual role of p53 in autophagy regulation. In the nucleus, p53 functions as a pro-autophagy agent through activation of AMP-activated protein kinase to inhibit mTOR phosphorylation. However, in the cytoplasm, p53 can inhibit autophagy in a transcriptionally non-dependent manner by suppressing glycolysis and reactive oxygen species (ROS) production (69). In addition to its involvement in the glycolytic pathway, p53 is also involved in the lipid metabolism of tumor cells. For example, p53 is capable of regulating the lipid metabolism of prostate cancer via the EXO1/p53/SREBP1 axis (70). In recent years, researchers have begun to investigate the potential of enhancing the sensitivity of NK cells to kill tumors by modulating the lipid metabolism of tumors. For example, Belkahla S (50) et al. found that dichloroacetic acid (DCA), which induces oxidative phosphorylation, was able to activate p53 to promote lipolysis in multiple myeloma cells, thereby enhancing the sensitivity of NK cells to tumor cell recognition and killing.

### 2.3 Regulation of the tumor microenvironment by p53

**Figure 1:**
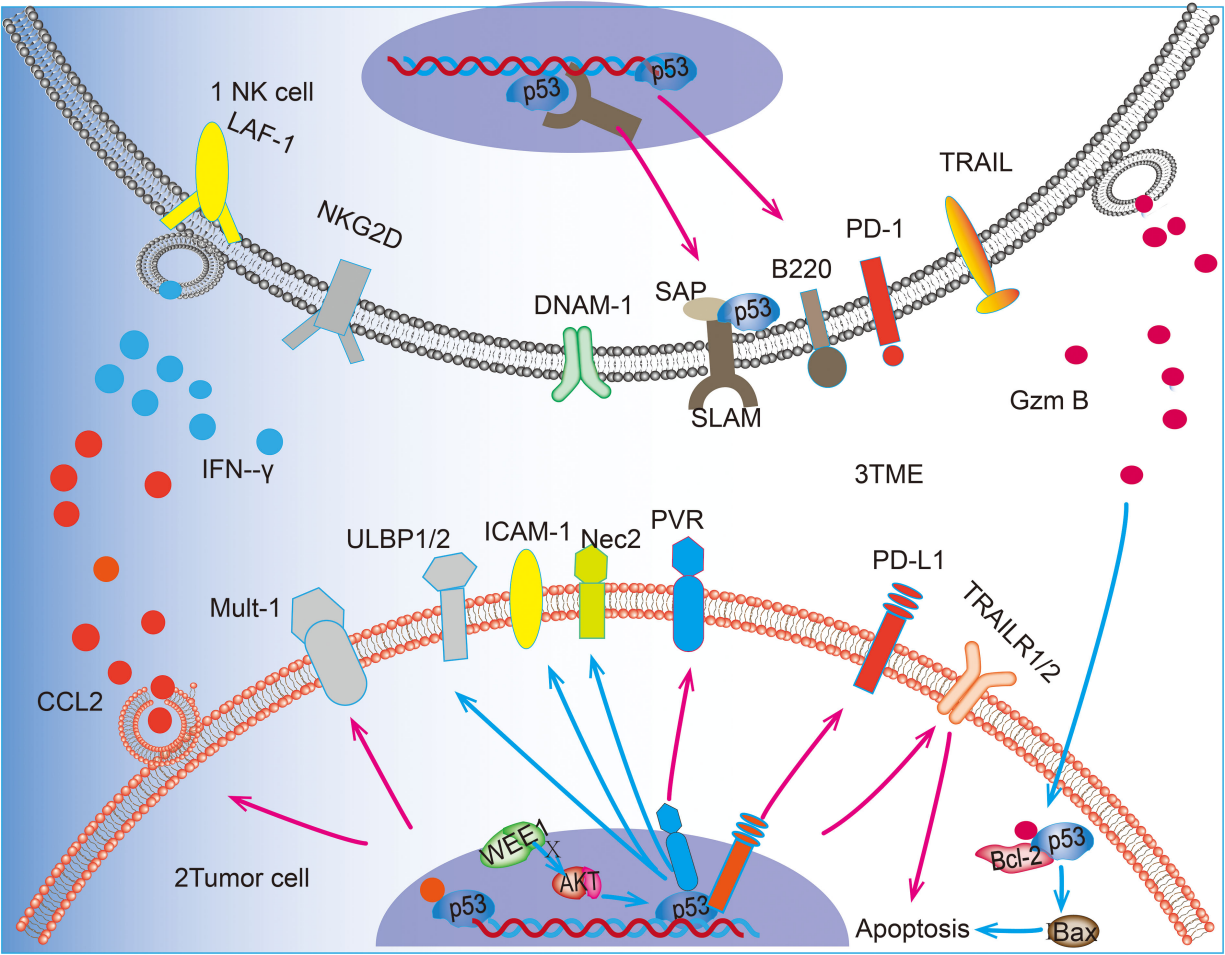
p53 can upregulate the expression of receptor ligands on the surface of NK cells and promote the killing and recognition of tumor cells by NK cells. 1 In NK cells, p53 can promote the transcription of SAP and up-regulate the expression of B220. 2 In tumor cells, p53 acts as a transcription factor for PVR and PD-L1 on the surface of tumor cells and up-regulates the expression of ULBP1/2 and Nec2 through the WEE1/AKT pathway. In addition, p53 was able to up-regulate the expression of Mult-1. p53 is involved in the pro-tumor apoptotic effect of GzmB released by N cells. p53 promotes BAX-mediated apoptosis by blocking the truncation produced by GzmB with Bcl-2, in addition to promoting the expression of death receptor TRAILR1/2 to promote apoptosis. 3 In the TME, p53 accelerates the recruitment of NK cells by promoting the release of CCL2 from tumor cells and also promotes the release of IFN-γ from NK cells, thus regulating the killing effect of NK cells tumor cells. 4Blue arrows(“**↑**”)represent the key steps or interactions within cellular signaling pathways. Pink arrows(“**↑**”) indicate the expression process of transcriptional regulatory proteins and how they affect the transcriptional activity of target genes.

TME refers to the non-tumor cells and components surrounding tumor cells, including immune cells, blood vessels, stromal cells, extracellular matrix, and lysogenic factors (71). Immune cells represent a crucial cellular component of the TME, and p53 plays a pivotal role in NK cells within the TME. Upon activation, p53 enhances the activation of NK cells in TME, thereby significantly reversing the immunosuppressed TME (72). NK cells, T cells and macrophages are members of the TME, and their interaction can optimize the antitumor effects of NK cells. Acetylation of p53 by p300/CBP acetyltransferase promotes macrophage polarization toward the M1 phenotype, which in turn enhances NK cell activity (20, 22, 73). p53 is instrumental in maintaining T cell homeostasis through SAP, and it can activate and recruit T cells, resulting in a notable increase in the proportion of CD8+ T cells in the TME when p53 is activated (19, 74). It has been demonstrated that activated T cells maintain the viability of NK cells and promote their antibody-dependent cytotoxicity (ADCC) effects (21). The communication between immune cells is facilitated by cytokines, which serve as a medium for transmitting interactions between cells. These factors play crucial roles in tumor immunosurveillance; chemokines attract rapidly migrating NK cells to tumors or inflammatory sites, while pro-inflammatory factors enhance immune responses against tumors by activating NK cell functions (75, 76). p53 can also regulate the production of these cytokines in the TME, and an experiment on a mouse model of hepatocellular carcinoma found that p53 activation promoted the promotion of the pro-inflammatory cytokines IL-12, IL-15, and IL-18 in the TME, and p53 is directly linked to the regulatory sequence of the CCL2 gene, p53 in hepatocellular carcinoma cells can recruit NK cells by inducing chemokine expression, thereby facilitating the elimination of tumor cells by NK cells (77). Many cytokines such as IFN-α2b and IL-2 can also regulate NK cell-mediated cytotoxicity by activating p53 (78). NK cells effectively remove senescent tumor cells through granule exocytosis, which further enhances the body’s immune defense against tumorigenesis (79). However, the infiltration of monocytes, neutrophils, and interstitial macrophages into the TME is induced by p53 reactivation in the context of lung adenocarcinoma. This accelerates the senescence response of lung adenocarcinoma cells. However, instead of accelerating clearance of lung adenocarcinoma, NK cells restrict the process of tumor elimination and promote senescence response after p53 reactivation. This may be due to the intensification of the immune-inflammatory response after p53 activation (33). Further investigation into the complex regulatory role of p53 in TME is warranted as it could provide impetus for studying broader mechanisms through which different cancer contexts regulate TME to promote tumor elimination. To highlight the role of p53, we have summarized the major roles of p53 in promoting NK cell tumor surveillance in NK cells, tumor cells, and the TME in **Figure 1**.

### 2.4 p53 mutations in tumor immune surveillance of NK cells

An additional explanation for the role of P53 in NK cell tumor immunosurveillance can be derived from the analysis of the impact of p53 mutations on NK cells in patients with different tumors. Although p53 functions as a key tumor suppressor, mutations of p53 are frequently observed in cancer patients. Unlike many other tumor suppressor genes, which are typically inactivated by deletion or truncating mutations in cancer, the majority of p53 mutations in cancer are missense mutations (80). Mutp53 frequently exhibits a pro-oncogenic phenotype, which is strongly associated with poor prognosis in cancer patients (81). In addition to losing wtp53 function in tumor suppression, mutp53 typically promotes tumor progression through a gain-of-function (GOF) mechanism (82). It has been demonstrated that a mouse breast cancer model with mutp53 exhibits GOF and has less NK cell infiltration and lower levels of secreted IFN-γ compared to mice expressing wtp53. The presence of p53 missense mutants in cancer cells impairs the role of NK cells and promotes cancer cell immune escape, thereby promoting breast cancer progression (44). The extracellular activity of NK cells in tumors expressing mutp53 is associated with mutations in the mutp53 cell lines. Furthermore, the status of the receptor on the surface of the NK cell affects p53 mutations, it has been identified that KIR is an NK cell inhibitory receptor and that B haplotype blocks of KIR correlate with mutations of p53. This has led to the conclusion that KIR B carriers have a higher likelihood of p53 mutations (p<0.004) and a higher risk of cancer (83). Mutp53 is also prevalent in patients with malignant NK-cell lymphomas, such as nasal NK-cell lymphoma and aggressive NK-cell leukemia, and the most common form of the mutation is also missense (84, 85). It has been demonstrated that mutp53 may be associated with Epstein-Barr virus (EBV) infection in patients with nasal NK cell lymphoma (86). Missense mutp53 is associated with the expression of the EBV-carcinogenic protein latent membrane protein type 1, which is associated with poor prognosis and lower survival in NK-cell lymphoma (87). Belkahla et al. (50) observed that mutp53 exerted an inverse effect on the MM1 cell line of MM when mutated and that the administration of the metabolic modulator DCA did not enhance the expression of NK-cell receptor-activating ligands, such as ULBP1, in the p53-mutant cell line. The mutant p53 missense mutant G242A, expressed on breast and colorectal cancer cells, has been observed to upregulate the inhibitory ligand H60a and downregulate the activating ligand for the NKG2D receptor, Mult-1. This results in an alteration in tumor recognition by NK cells, which is the opposite of the effects of wtp53 described above (44). Mutations in p53 are more prone to the formation of tumor-supporting TME. It has been demonstrated that colorectal cancer cells harboring mutp53 exhibit GOF, and tumor cells secrete miR-1246 to convert macrophages to a pro-carcinogenic M2 phenotype, increasing the infiltration of M2-type pro-carcinogenic macrophages in the TMEs (88). In contrast, M2-type macrophages have been demonstrated to limit the function of NK cells (89), which is also contrary to the aforementioned role of p53 in promoting the function of M1-type macrophages and thus the tumor-killing role of NK cells (22). The demonstration of the oncogenicity of p53 mutations in NK cell malignancies provides insight into the role of mutp53 in NK cell tumor killing and NK cell malignancies, which in turn facilitates a deeper understanding of the role of p53.

## 3 p53-a potential target for enhancing immune checkpoint inhibitor therapy

Immune checkpoint inhibitors have emerged as a successful immunotherapeutic approach for cancer treatment. These inhibitors target the dysfunctional immune system, particularly antibodies such as anti-CTLA4 and anti-PD-1, to induce immune cells to kill cancer cells. Studies have shown that these antibodies can improve the outcome and prognosis of cancer patients across various types of cancers. This treatment has been effective in treating a variety of cancers (90, 91). The p53 pathway plays a crucial role in regulating biological processes, and modulation of this pathway in cancer cells expressing wtp53 has been found to enhance NK cell-driven immunotherapeutic responses. The researchers discovered that p53-activated NK cells were capable of activating the immune response, transforming melanomas that were unresponsive to anti-PD-1 antibody treatment into tumors that were responsive to anti-PD-1 antibody treatment, thereby altering the immune status from “cold” to “hot” (18). Deng et al. (92) have demonstrated that p53 can impede the progression of triple-negative breast cancer by negatively regulating the expression of PD-L1. Additionally, p53 can be employed as a target for tumor immunotherapy, with the consequence that targeting p53 can enhance the sensitivity to anti-PD-1 therapy. MDM2 is a negative regulator of p53, and DS-5272 is an inhibitor targeting MDM2-p53 (93). DS-5272 has been found capable of activating p53-induced up-regulation of active markers (CD107a and IFN-γ) in NK cells while inhibiting NK cell-mediated cytotoxicity through activation of the HIF1α-PD-L1 pathway. This approach proves beneficial in treating acute myeloid leukemia (AML), where combining DS-5272 with anti-PD-1 enhances the efficacy of anti-PD-1 treatment in AML cases. The treatment strategy for AML has shown improved sensitivity toward anti-PD-1 therapy (94).

B7-H3, along with PD-L1 and CTLA4, is a member of the B7 protein family and is a promising immune checkpoint for antibody-based immunotherapy (95). B7-H3 is highly expressed in tumor cells, tumor-associated vasculature systems, and stroma (96). Inhibition of B7-H3 enhances NK cell cytotoxicity, characterized by increased production of GzmB, IFN-γ, and IL-2 (97). Deficiency of p53 and PTEN expression induces B7-H3 overexpression in cancer cells, and their study demonstrated that B7-H3 inhibitors, in combination with antibodies such as anti-CTLA4 and anti-PD-1, showed high efficacy in a p53/PTEN-deficient mouse model of prostate cancer. The treatment resulted in a durable antitumor effect with curative potential and was able to enhance the sensitivity of anti-CTLA4 and anti-PD-1 therapy (25). It is important to acknowledge that the impact of immune checkpoint inhibitors on different types and stages of cancer varies. Some patients may not respond to the treatment or develop drug resistance. Additionally, therapies targeting p53 may encounter challenges related to drug toxicity and side effects in clinical practice. p53-regulated immune checkpoints may exhibit varying expression levels and functions in different cancers. Further research is needed to elucidate precise regulatory mechanisms and optimal utilization of these pathways.

## 4 Other therapies for enhancing NK cell-mediated tumor sensitivity through p53

In contrast to T cells and B cells, NK cells have the unique ability to directly recognize and kill tumor cells without requiring antigen presentation (98). Unlike T cells, NK cells do not possess surface T-cell receptors (TCRs) and, therefore, do not cause cytokine release syndrome (CRS) or graft-versus-host disease (GVHD). This makes NK cell-based immunotherapy a promising avenue for further research (99). Clinical trials have been conducted on chimeric antigen receptor-modified NK cell (CAR-NK) therapies for tumor treatment. These therapies have demonstrated improved efficacy by enhancing the recognition of specific molecules expressed on the surface of tumor cells (9). The sensitivity of CAR-NK therapy can be further enhanced through p53-based therapies.

Nutlin-3a, a small molecule known to inhibit MDM2, has been shown to activate the p53 function effectively (100). In their investigation, Focaccetti et al. (26) treated neuroblastoma cells with Nutlin-3a and observed increased sensitivity to CAR-NK cell therapy targeting DNAM-1. Furthermore, a combination of local radiofrequency ablation (RFA) and melatonin (MLT) in lung cancer patients resulted in the upregulation of the p53 signaling pathways and significantly enhanced anti-tumor immunity mediated by NK cells (101). Strobel et al. (102) identified potential targets for CAR-NK therapy in melanoma through immunohistochemistry, revealing that p53 was overexpressed in 37% of melanoma patients. However, due to the numerous mutations across different exons of p53 (14), further investigation into direct targeting of p53 for CAR-NK therapy is warranted. The PARP inhibitor talazoparib has shown efficacy as an anticancer drug by promoting PARP1 binding and inducing tumor cell senescence through inhibition of p53 ubiquitination. When combined with other anti-aging drugs, it has been demonstrated to enhance the infiltration of tumor NK cells and T cells within the TME, leading to a more anti-tumorigenic state (103). Studies have shown that Sijunzi Decoction (SJZD), a traditional Chinese herbal medicine, significantly inhibits mouse colorectal cancer growth. Additionally, SJZD increases colon cancer cell sensitivity to natural killer (NK) cell-mediated killing by modulating p53 expression levels and enhancing death receptor 4 (DR4) and death receptor 5 (DR5). This mechanism ultimately enhances colon cancer cell susceptibility to NK cell-mediated killing (63). While NK cells and p53 hold potential in tumor therapy, there are still challenges and limitations in targeting p53 in NK cells for tumor treatment. Although current research has explored treatments for mutp53 in malignant NK cell tumors, such as the combination of ruxolitinib with the farnesyltransferase inhibitor tipifarnib targeting p53-mutated NK cell malignancies (104). The efficacy of STING agonists alone or in combination with vinotec has been demonstrated in both p53-mutated AML patient samples and PDX models (105). Mutp53 may lead to its loss or alteration of function, which could impact the therapeutic efficacy of NK cells. In addition, due to the different effects of p53 on NK cell regulation in different tumors, a single agent may not be able to address the complexity and heterogeneity of tumors. While combination therapies may enhance the anti-tumor immune response of NK cells, the safety, efficacy, and long-term outcomes of such treatments require further investigation.

## 5 Conclusion and outlook

The p53 protein is involved in multiple aspects of tumor progression. Its activation not only inhibits the advancement of solid and hematological tumors but also enhances the recognition and elimination of tumor cells by NK cells (94, 106). This process involves multiple mechanisms through which p53 influences the activity and function of NK cells, ultimately leading to antitumor effects. In NK cells, p53 plays a role in regulating the balance of the NK cell cycle and apoptosis. Additionally, it has been demonstrated to promote the expression of NK cell surface receptors, thereby enhancing NK cell activation (30, 33). Furthermore, p53 has been shown to augment NK cell activity by upregulating NK cell-activating receptors and ligands. The up-regulation of these receptors and ligands enhances the interaction between NK cells and tumor cells, which in turn promotes NK cell-mediated tumor cell killing (15, 18). Furthermore, p53 can also affect NK cell activity by regulating the TME. The TME plays a key role in tumor development and therapeutic response, and p53, as one of the regulators of TME, interacts with components of TME, thereby affecting NK cell activity and function (72). Moreover, p53 participates in diverse signaling pathways that influence the ability of NK cells to kill tumors by regulating apoptosis and autophagy in tumor cells (16, 55). It is noteworthy that the role of p53 in NK cells is not solely promotional. The aforementioned study demonstrated that NK cells do not participate in the elimination of lung adenocarcinoma when p53 is reactivated despite the overexpression of the activation receptor B220 on the surface of NK cells under the regulation of p53 (33). Mutations of p53 are frequently observed in tumor cells, resulting in mutated forms with GOF properties that exert effects opposite to those mediated by wtp53. These effects can impact immune surveillance against tumors by NK cells (44, 80). Understanding this complex relationship between p53 and NK cells provides a promising avenue for developing innovative cancer therapies.

Despite decades of efforts to develop p53-related drugs, concerns regarding the therapeutic benefits of such interventions have persisted. However, recent years have witnessed a gradual refutation of this perspective, as emerging evidence substantiates the druggability of p53 (14). Consequently, there is an increasing call for expediting the translation of p53-based cancer therapies into clinical treatment strategies (107). Many small molecule drugs targeting p53 have entered clinical trials. These drugs primarily recognize and intervene in the interactions between p53 and its negative regulators, with MDM2 representing the most notable negative regulator. For instance, APG-115, a potent MDM2 inhibitor with high oral bioavailability, demonstrated potent antitumor effects in preclinical models of AML (108). In cancer treatment settings, combining NK cell-based therapy with targeted p53 therapy can enhance therapeutic efficacy while reducing drug resistance. Furthermore, integrating targeted p53 drugs with immunotherapies like immune checkpoint inhibitors or CAR-NK can augment human immune system aggressiveness against tumors and thereby increase immunotherapy sensitivity (18, 26). Moreover, research on novel drugs or drug delivery systems has become abundant in recent years. Zhang et al. (58), for example, developed an all-in-one peptide capable of reversing TME-induced immunosuppression through activating p53. Additionally, a dual-targeted mRNA nano delivery system based on cationic lipids and hyaluronic acid has been devised to deliver therapeutic p53 mRNA along with other mRNAs to tumor TMEs; this approach enhances anti-tumor immunity and effectively inhibits lung cancer progression (109). Consequently, the activation or reactivation of p53 represents a promising strategy to enhance NK cell activity and tumor-killing susceptibility, a line of inquiry worthy of continued investigation. It is important to recognize that targeting p53 in the immune system may have complex systemic effects, and that p53 mutations and drug resistance are inevitable in patients with different tumors. In conclusion, the activation or reactivation of p53 represents a promising strategy to enhance NK cell activity and tumor killing susceptibility. However, this approach must be carefully evaluated in different tumor types and biological contexts.
